# Supplementary material for: CRISPR activation screens identify core protein-dependent regulation of heparan sulfate sulfation and ligand specificity
Source: bioRxiv. 2026 Jun 30:2026.06.29.735380. Preprint. [Version 1] doi: 10.64898/2026.06.29.735380 (PMC13345059; doi:10.64898/2026.06.29.735380)
Supplement: Supplement 1 [file media-1.pdf]

Supplementary Information for:

CRISPR activation screens identify core protein-dependent control of heparan sulfate sulfation and ligand specificity

Jack C. Moore[1,2], Haruki Takeuchi[1,2#], Caitlien Nguyen[1#], Chin Huang[1,2], Digantkumar Chapla[1], Amrita Basu[1], Zhangjie Wang[3], Jian Liu[4], Kelley W. Moremen[1,2], Ryan J. Weiss[1,2*]

*Correspondence should be addressed to R.J.W.
Email: ryan.weiss@uga.edu

This PDF file includes:

Supplementary Tables 1-6
Supplementary Figures 1-4

Supplementary Table 1:
HS disaccharide composition for HS chains isolated from recombinant eSDC1 and eSDC2

**HS Disaccharides**		**Abundance (% Total Disaccharide)**	
**Structure Code**[a]	**Unit Formula**[b]	**eSDC1 (% Total HS)**	**eSDC2 (% Total HS)**
D0A0	ΔUA-GlcNAc	69.20 ± 1.04	71.44 ± 0.60
D2A0	ΔUA2S-GlcNAc	1.13 ± 0.03	0.82 ± 0.02
D0A6	ΔUA-GlcNAc6S	2.40 ± 0.09	1.19 ± 0.14
D2A6	ΔUA2S-GlcNAc6S	0.10 ± 0.02	0.05 ± 0.00
D0S0	ΔUA-GlcNS	14.73 ± 0.68	14.71 ± 0.26
D2S0	ΔUA2S-GlcNS	9.59 ± 0.61	10.13 ± 0.46
D0S6	ΔUA-GlcNS6S	2.01 ± 0.06	1.20 ± 0.11
D2S6	ΔUA2S-GlcNS6S	0.84 ± 0.21	0.46 ± 0.10

[a] The disaccharide structure code is described in (Lawrence, et al. *Nat. Methods* 2008)
[b] ΔUA = 4,5-unsaturated uronic acid

Supplementary Table 2:
Distribution of HS disaccharides for HS chains isolated from recombinant ectodomain SDC1 (eSDC1) and SDC2 (eSDC2)

**HS Sulfates/disaccharide**	**Abundance (% Total Disaccharide)**	
	**eSDC1**	**eSDC2**
N-Ac	73.71 ± 1.14	73.88 ± 0.46
N-SO_3	25.15 ± 1.11	25.30 ± 0.46
2-*O* SO_3	13.67 ± 0.50	12.66 ± 0.47
6-*O* SO_3	5.35 ± 0.35	2.90 ± 0.15

Supplementary Table 3:
HS tetrasaccharide composition for HS chains isolated from recombinant eSDC1 and eSDC2

**HS tetrasaccharides**		**Abundance (pg / mg protein)**	
**Structure Code**[a]	**Unit Formula**[b]	**eSDC1**	**eSDC2**
D0N6-G0S9	ΔUA-GlcNAc6s-GlcA-GlcNS3S6S	4.75 ± 0.50	1.25 ± 0.50
D0S6-G0S9	ΔUA-GlcNS6S-GlcA-GlcNs3S6S	18.25 ± 4.50	7.25 ± 5.25
D0S6-I2S9	ΔUA-GlcNS6S-IdoA2S-GlcNS3S6S	9.25 ± 2.87	8.5 ± 1.29
D0S0-I2S3	ΔUA-GlcNS-IdoA2S-GlcNS3S	79.50 ± 5.80	109.50 ± 6.45
D2S0-I2S3	ΔUA2S-GlcNS-IdoA2S-GlcNS3S	53.50 ± 14.55	78.50 ± 9.85

[a] The tetrasaccharide structure code is described in (Lawrence, et al. *Nat. Methods* 2008)
[b] ΔUA = 4,5-unsaturated uronic acid

Supplementary Table 4:
CS/DS disaccharide composition for CS/DS isolated from recombinant eSDC1 and eSDC2

CS/DS Disaccharides		Abundance (% Total Disaccharide)[c]	
**Structure Code[a]**	**Unit Formula[b]**	**eSDC1 (% Total CS)**	**eSDC2 (% Total CS)**
D0a0	ΔUA-GalNAc	11.12 ± 0.04	18.77 ± 0.09
D0a4	ΔUA2S-GalNH$_2$	51.61 ± 0.27	49.26 ± 0.23
D0a6	ΔUA-GalNS	36.78 ± 0.27	31.66 ± 0.31
D2a10	ΔUA-GalNAc6S	0.50 ± 0.00	0.31 ± 0.00

[a] The disaccharide structure code is described in (Lawrence, et al. *Nat. Methods* 2008)
[b] ΔUA = 4,5-unsaturated uronic acid

Supplementary Table 5:
sgRNA sequences used in this study

Gene Target	Forward guide sequence (5'-3')	Reverse guide sequence (3'-5')
HS3ST1	AGGGAGAGCGCGTTGGGCAG	CTGCCCAACGCGCTCTCCCT
SDC1	GGCGTTCCGAAGGGGCCGGG	CCCGGCCCCTTCGGAACGCC
SDC2	AGAAGCAGGCTCAGGAGGGA	TCCCTCCTGAGCCTGCTTCT
SDC3	GCGGCGGCGCACGCTTCCTG	CAGGAAGCGTGCGCCGCCGC
SDC4	CCCCGCCCGGAATTCCCCAG	CTGGGGAATTCCGGGCGGGG

Supplementary Table 6:
Primer sequences used for quantitative PCR experiments

Gene	Forward primer sequence (5'-3')	Reverse primer sequence (5'-3')
YWHAZ	CCTGCATGAAGTCTGTAACTGAG	GACCTACGGGCTCCTACAACA
SDC1	ACGAAGGCAGCTACTCCTTG	GTTTGGTGGGCTTCTGGTAG
SDC2	GCTGTTGGTGTATCGCATGA	ACTGGATGGTTTGCGTTCTC
SDC3	GCCACCACTGCTGTTATAAGG	ACTGTGGTCAGTGGGAGAGG
SDC4	ACTGTGGTCAGTGGGAGAGG	GCTGCCTTCATCCTTCTTCTT
HS3ST1	AACGAGGTCCACTTCTTCGA	GCATCTGGCTGAGGTACCAG
HS3ST3A1	CGGAAGTTCTTGCTGATGCT	CTCGGCCAGGCAGTAGAA
HS3ST3B1	CCGGTGAGGAGGAAGCTC	GGCGCACGAGTACAGGAA
HS3ST5	CAATTTCATGTCGTCGATGG	AGGAACTTCTCCACGAGCTG
NDST1	TCCTGCTGTTCATCTTCTGC	CTCGCTTCCAGCCATATAGG
HS6ST2	TACACTGGCGATGACTGGTC	GCGGTTGTTGGCTAGATTGT

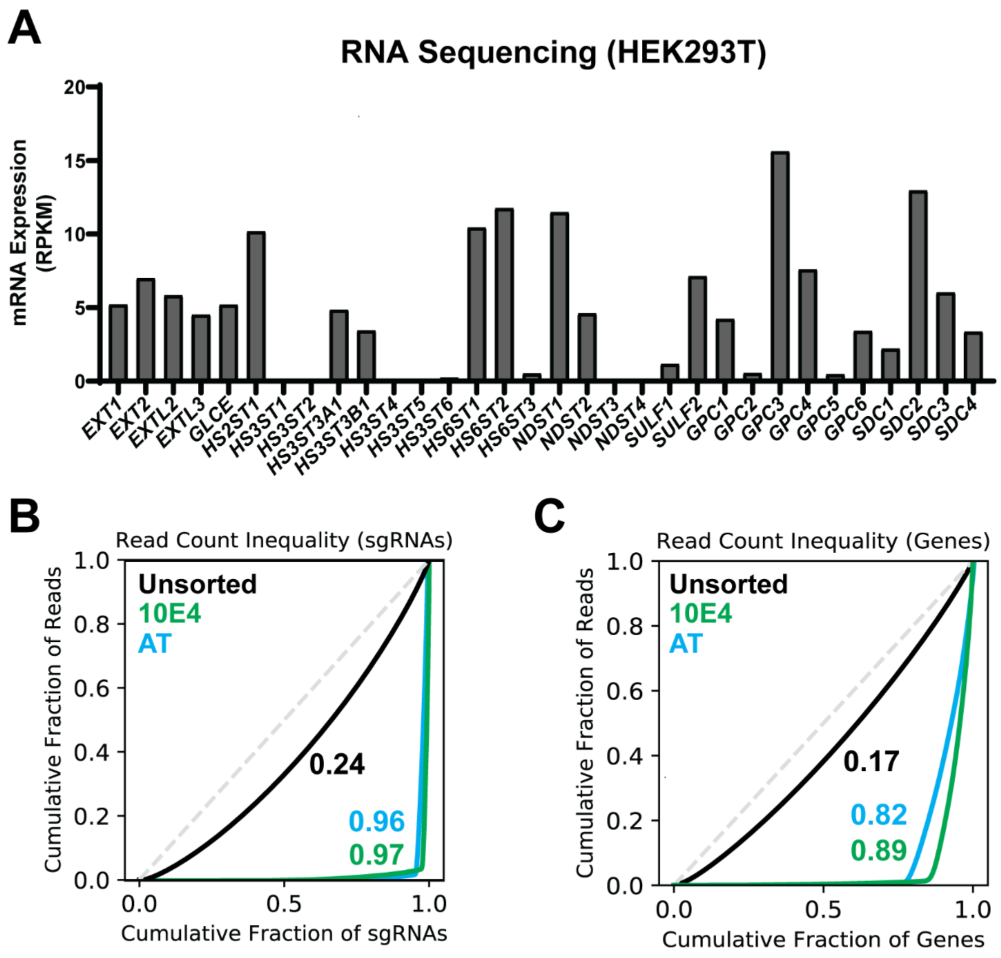

Supplementary Figure 1. HEK293T RNA sequencing data for HSPG genes and CRISPRa screening read distributions. (A) RNA Sequencing of HEK293T WT cells. RPKM values shown represent an average of biological duplicates (n = 2 independent biological replicates). Lorenz curves showing the distribution of **(B)** sgRNA and **(C)** gene sequencing reads in unsorted control cells versus 10E4-, and AT-sorted cell populations. Numbers represent Gini coefficients (0: reads cover sgRNA/gene library evenly, 1: reads cover only a single sgRNA/gene).

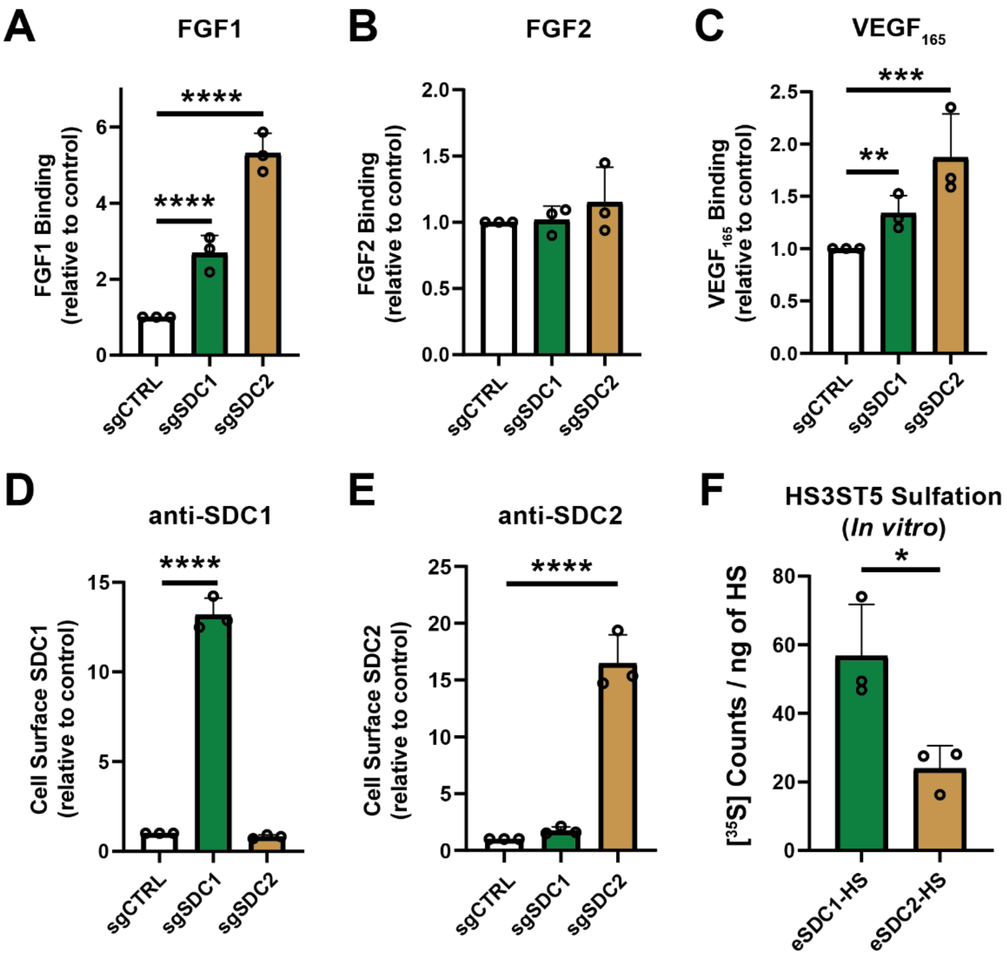

Supplemental Figure 2. Characterization of SDC1 and SDC2 CRISPRa cell lines. Flow cytometry analyses of **(A)** FGF1, **(B)** FGF2, and **(C)** $VEGF_{165}$ cell surface binding for SDC1 and SDC2 activation lines compared to sgCTRL cells. **(D-E)** Flow cytometry analyses of cell surface SDC1 and SDC2 levels in activation lines versus control cells. **(F)** $[^{35}S]$ counts normalized to HS mass input for in vitro HS3ST5 sulfation reactions. Data are presented as mean ± SD (n = 3 independent experiments), *****p*<0.0001, ****p*<0.001, ***p*<0.01, **p*<0.05 by two-sided t-test.

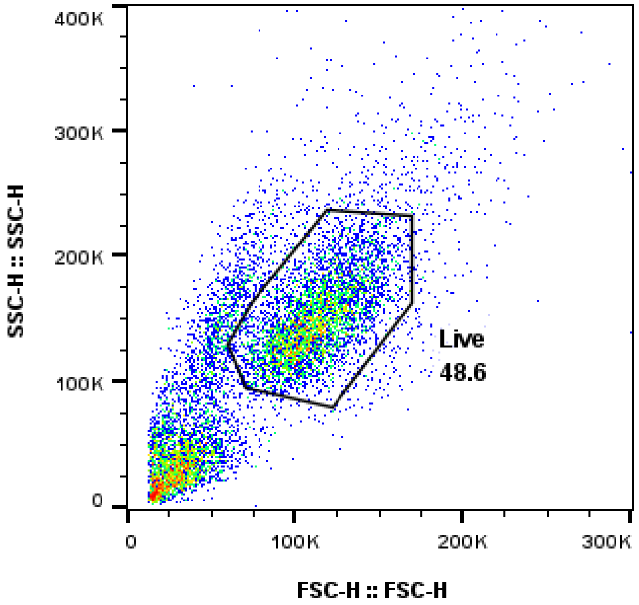

Supplementary Figure 3. General flow cytometry gating strategy. Cells were gated based on forward and side scattering for analysis of flow cytometry data.

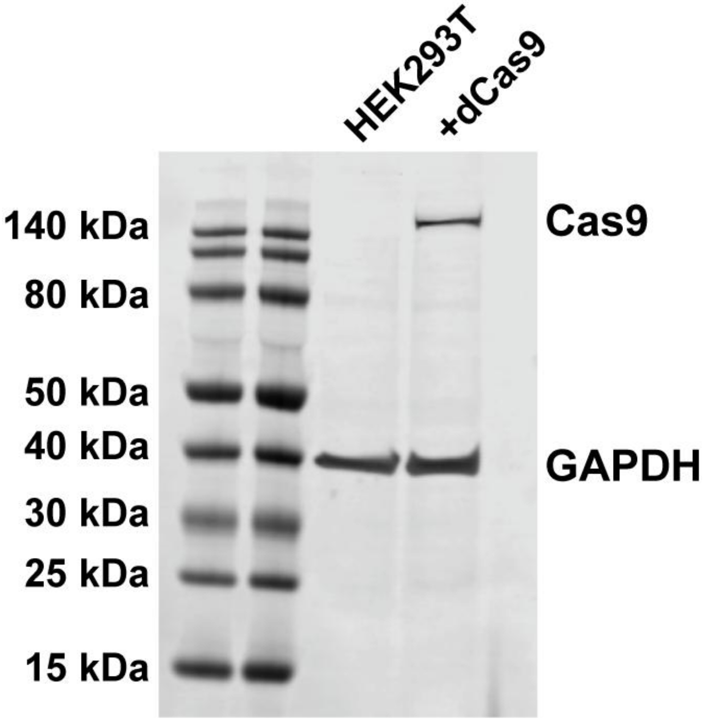

Supplementary Figure 4. Source Data. Uncropped western blot source image for Figure 1B.
